# Protective effects of nattokinase against microvasculopathy and neuroinflammation in diabetic retinopathy

**DOI:** 10.1111/1753-0407.13439

**Published:** 2023-07-04

**Authors:** Zijing Huang, Wai Kit Chu, Tsz Kin Ng, Shaolang Chen, Jiajian Liang, Chong‐Bo Chen, Yanxuan Xu, Biyao Xie, Shuping Ke, Qingping Liu, Weiqi Chen, Dingguo Huang

**Affiliations:** ^1^ Joint Shantou International Eye Center of Shantou University and The Chinese University of Hong Kong Shantou China; ^2^ Department of Ophthalmology & Visual Sciences The Chinese University of Hong Kong Hong Kong China; ^3^ Shantou University Medical College Shantou China

**Keywords:** diabetic retinopathy, high mobility group box 1, microvasculopathy, nattokinase, neuroinflammation, 糖尿病视网膜病变, 纳豆激酶, 微血管病变, 神经炎症, 高迁移率族蛋白B1

## Abstract

**Aims:**

Diabetic retinopathy (DR) is a significant global public health concern. Alternative, safe, and cost‐effective pharmacologic approaches are warranted. We aimed to investigate the therapeutic potential of nattokinase (NK) for early DR and the underlying molecular mechanism.

**Methods:**

A mouse model of diabetes induced by streptozotocin was utilized and NK was administered via intravitreal injection. Microvascular abnormities were evaluated by examining the leakage from blood–retinal barrier dysfunction and loss of pericytes. Retinal neuroinflammation was examined through the assessment of glial activation and leukostasis. The level of high mobility group box 1 (HMGB1) and its downstream signaling molecules was evaluated following NK treatment.

**Results:**

NK administration significantly improved the blood–retinal barrier function and rescued pericyte loss in the diabetic retinas. Additionally, NK treatment inhibited diabetes‐induced gliosis and inflammatory response and protected retinal neurons from diabetes‐induced injury. NK also improved high glucose‐induced dysfunction in cultured human retinal micrangium endothelial cells. Mechanistically, NK regulated diabetes‐induced inflammation partially by modulating HMGB1 signaling in the activated microglia.

**Conclusions:**

This study demonstrated the protective effects of NK against microvascular damages and neuroinflammation in the streptozotocin‐induced DR model, suggesting that NK could be a potential pharmaceutical agent for the treatment of DR.

## Graphical abstract

Highlights

- Nattokinase rescued endothelial dysfunction and pericyte loss in the diabetic retina.
- Nattokinase attenuated diabetes‐induced retinal inflammation and neurodegeneration.
- High mobility group box 1 signaling was involved in nattokinase‐mediated retinal protection.
- Nattokinase might be developed as a pharmaceutical product for early diabetic retinopathy.

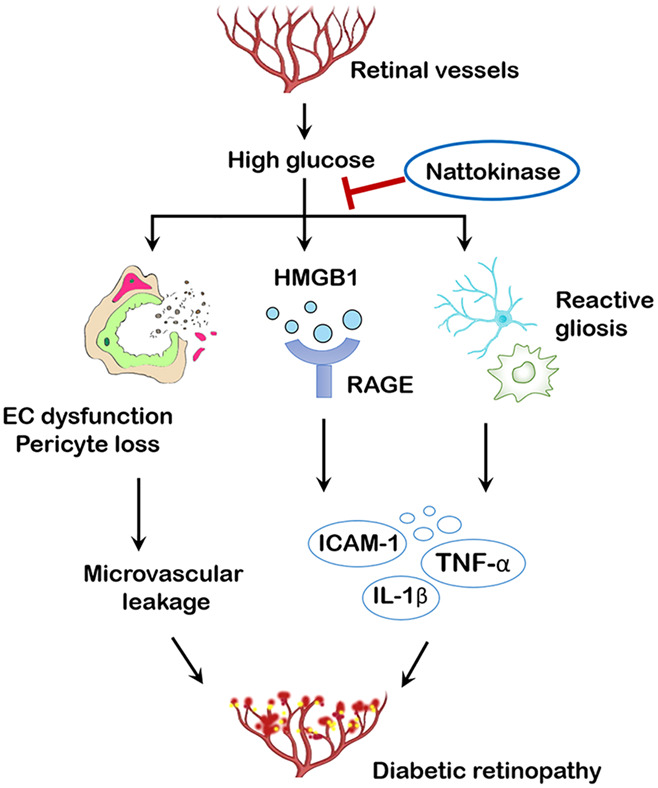

## 摘要

目的: 糖尿病视网膜病变（DR）是一个重要的全球公共卫生问题。需要寻求安全、有效且经济的替代性药物治疗方案。本研究的目的是探讨纳豆激酶（NK）对早期DR的治疗潜力及其潜在的分子机制。

方法: 使用链脲佐啶诱导的糖尿病小鼠模型，通过玻璃体腔内注射给予NK。通过检查血液视网膜屏障功能障碍导致的渗漏和毛细血管周细胞丢失来评估微血管异常。通过评估胶质细胞活化和白细胞粘附来评估视网膜神经炎症。在NK处理后，评估高迁移率族蛋白B1（HMGB1）及其下游信号分子的水平。

结果: NK处理显著改善了糖尿病大鼠血‐视网膜屏障功能并逆转了糖尿病视网膜中毛细血管周细胞的丢失。此外，NK处理抑制了糖尿病诱导的胶质细胞活化和炎症反应，并保护视网膜神经元免受糖尿病损伤。NK还改善了高糖诱导的人视网膜微血管内皮细胞功能障碍。在机制上，NK通过调节活化的小胶质细胞中的HMGB1信号，部分调节糖尿病诱导的炎症。

结论: 本研究表明，NK对链脲佐啶诱导的DR模型中的微血管损伤和神经炎症具有保护作用，这表明NK可能是治疗DR的潜在药物。

## 1 INTRODUCTION

Diabetic retinopathy (DR), the most common diabetic microvascular complications in the eye, is the leading cause of irreversible blindness among the working‐age populations.^1^ The incidence of DR increases with the duration of diabetes despite intensive blood glucose control.^2^ Although antivascular endothelial growth factor (VEGF) agents have been approved as a first‐line therapy for DR and diabetic macular edema, there are concerns about drug resistance and no response in some DR patients, macular ischemia and scarring, and recurrence.^3^, ^4^ Therefore, exploration of alternative treatments is warranted.

One of the hallmarks in early‐stage DR is the loss of retinal pericytes.^5^, ^6^, ^7^ These specialized contractile mesenchymal cells are located beneath and within the capillary basement membrane and possess multiple vasoregulatory activities. The presence of abundant contractile proteins and myosin enables the pericytes to precisely regulate the vascular tone, perfusion pressure, and permeability.^8^ Importantly, healthy pericytes are essential in supporting the survival, growth, and differentiation of endothelial cells, which play a pivotal role in the maintenance of the blood–retinal barrier (BRB).^8^, ^9^ Targeting pericyte loss has shown great therapeutic potential in early‐stage DR.^10^ The development of pericyte targeting drugs is highly necessary.

Chronic low‐grade inflammation also contributes to pericyte loss and breakdown of BRB in DR.^11^, ^12^ High glucose (HG) insults trigger the activation of multiple inflammatory cytokines and chemokines, among which high mobility family protein 1 (HMGB1) is a key mediator of inflammatory and autoimmune disorders.^13^, ^14^, ^15^, ^16^ In the diabetic retina, HMGB1 interacts with toll‐like receptor 4 (TLR4) and the receptor for advanced glycation end‐products (RAGE), initiating the inflammatory cascade.^17^ HMGB1‐based therapies, using specific inhibitors or siRNA, have been shown to regulate BRB functions and rescue the dying vascular cells in diabetic animals,^18^, ^19^ indicating a promising target for DR treatment.

Nattokinase (NK) is an alkaline serine protease extracted from the traditional food natto. It has been used for preventing and treating cardiovascular disorders due to its pharmacologic properties, including anticoagulation, thrombolysis, antiatherosclerosis, and antihypertension.^20^ NK owns unique superiority over synthetic chemicals due to its natural sources, oral availability, safety, and fewer side effects.^21^, ^22^ Recently, NK supplements have shown to improve neural survival and reduce inflammatory responses in neurodegenerative animals,^23^, ^24^ suggesting its anti‐inflammatory and neuroprotective properties. Whether NK can protect the retina from diabetic vascular injury and inflammation requires further investigations.

In this study, we aimed to evaluate the treatment effects of NK against microvascular leakage in DR using a streptozotocin (STZ)‐induced diabetes model and a culture model of HG‐challenged retinal vascular cells. The changes in pericytes and the HMGB1‐related inflammation upon the NK treatment are also investigated.

## 2 MATERIALS AND METHODS

### 2.1 Ethics approval

The animal study was carried out in accordance with the ARRIVE (Animal Research: Reporting of In Vivo Experiments) guidelines and with the United Kingdom Animals (Scientific Procedures) Act, 1986 and the associated guidelines, and was approved by the Animal Ethic Committee of Joint Shantou International Eye Center of Shantou University and The Chinese University of Hong Kong. The mice were anesthetized with intramuscular injection of zoletil (35 mg/kg) and xylazine (10 mg/kg). The adequacy of anesthesia was confirmed by the absence of reflex response to foot squeeze. Body temperature was maintained at 37 ± 0.5°C during surgery. At the end of the experiments, the mice were euthanized through carbon dioxide asphyxiation of air with 100% carbon dioxide, within 6 min.

### 2.2 Streptozotocin‐induced diabetic retinopathy murine model

C57BL/6J mice were purchased from Beijing Vital River Laboratory Animal Technology Co., Ltd. (Beijing, China). The mice were maintained in a specific pathogen‐free facility. The STZ‐induced DR model was established as previously described.^25^ Briefly, the mice at 8 weeks of age were fasted overnight and then received intraperitoneal injections of STZ (50 mg/kg, Sigma‐Aldrich) diluted in 10 mM sodium citrate buffer for 5 consecutive days. Blood glucose levels were measured with an automatic analyzer (Accu‐Chek @Active, Roche) in blood samples from the tail vein. The mice with blood glucose levels maintained at over 16.6 mmol/L (300 mg/dL) were considered as diabetic. For the treatment, NK (Abmole Bioscience, Houston, TX; 1 μL of 1 μM, resolved in 10% [v/v] DMSO in phosphate buffer saline [PBS]) or vehicle (PBS containing equivalent DMSO) were administered via intravitreal injection bimonthly from the first to the fifth month after STZ induction (Figure S1A). For the mechanism investigation, intravitreal injection of recombinant human HMGB1 (1 μg in 1 μL; R&D Systems) or glycyrrhizin (1 μL of 100 μM; Sigma) was performed bimonthly from the first to the fifth month after STZ induction. At the end of the sixth month, the mice were euthanized, and the retinas were collected for further analyses.

### 2.3 Evans blue assay

Evans blue assay was performed at the end of the sixth month after STZ induction. The thoracic cavity was opened to expose the heart. The right atrium was cut with microscissors to stop blood returning from the lungs. One ml of Evans blue dye (50 mg/kg, molecular weight: 960.8, dissolved in PBS; Sigma‐Aldrich) was injected into the left ventricle.^26^ The mice were sacrificed, and the eyeballs were enucleated after the presence of a blue color appearance on the paws. The eyeballs were then fixed in 4% buffered paraformaldehyde for 30 min. The retinas were removed and cut radially cuts in the peripheral retina to allow the whole retina flat‐mounting on the glass slides. The Evans blue dye extravasation was detected in the retina (*n* = 9 retinas from 9 mice) with a confocal microscope (Carl Zeiss LSM700‐ZEN 2009, Germany). Vascular permeability was observed in different locations (central, midperipheral, and peripheral retina). The permeability of retinal microvessels was calculated as the percentage of leakage area to the total area from four images in the central, midperipheral, and peripheral retina. The areas of vascular leakage were assessed using Image‐Pro Plus 6.0 (Media Cybernetic, Rockville, MD). Other retinas (*n* = 9 retinas from 9 mice) were incubated with formamide overnight to extract Evans blue dye according to previously described methods.^26^ Absorbance of the extracts at 620 nm were detected using a microplate reader. The concentrations were calculated from a standard curve and normalized against the time‐averaged Evans blue plasma concentration and retina weight. The calculated Evans blue values were used for statistical analysis.

### 2.4 Fluorescein isothiocyanate (FITC)‐dextran assay

The periorbital area of the right eye was gently exposed. FITC‐dextran (50 mg/mL in 0.05 mL, molecular weight: 2000; Sigma‐Aldrich) was injected into the mouse's orbital venous sinus using a 27‐gauge needle with a 1 mL syringe.^27^ The eyeballs were enucleated at 5 min after injection, and the whole‐mount retina was carefully removed for confocal imaging. The vascular leakage of retinal microvessels was calculated as the percentage of leakage area to the total retina area (*n* = 6 retinas in each group).

### 2.5 Immunofluorescence assay

For retinal cryosections, the eyeballs were embedded in optimal cutting temperature compound at −20°C overnight. 8‐μm serial cryosections were prepared. The slices were probed with primary antibodies (1:100) at 4°C overnight followed by the incubation with fluorescence‐labeled secondary antibodies (1:1000, 1 hur) and counterstained with 4′6‐diamidino‐2‐phenylindole (DAPI, 1:1000, 5 min). For retinal flat mounts, eyeballs were fixed in 4% paraformaldehyde for 30 min and the retinas carefully were dissociated. After incubated with primary (1:100, 4°C, overnight) and respective secondary antibodies (1:1000, 1 h). The retinas were washed extensively and flat mounted on the slides. The primary antibodies included CD31 antibody (BD Bioscience, San Jose, CA, 550274), nerve/glial antigen 2 (NG2) antibody (Santa Cruz Biotechnology, Dallas, TX, sc‐166 251), ionized calcium‐binding adapter molecule 1 (Iba1) antibody (Wako Chemicals, Osaka, Japan, 019‐19 741), glial fibrillary acidic protein (GFAP) antibody (Abcam, Cambridge, UK, ab302644), HMGB1 antibody (Santa Cruz, sc‐74 085), β‐tubulin antibody (Abcam, ab215037), protein kinase C‐α antibody (Abcam, ab32376), and microtubule‐associated protein 2 antibody (Abcam, ab254264). The samples were imaged using a confocal microscope (Carl Zeiss LSM700) or an automated upright fluorescence camera (Leica DM4000B, Germany).

### 2.6 Retinal leukostasis assay

The retinal leukostasis assay was performed at the end of the sixth month after STZ induction. according to a previous report.^28^ The mice were anesthetized with zoletil and xylazine before their chest cavity was opened. A 20‐gauge perfusion cannula was inserted into the left heart ventricle. The right atrium was cut with microscissors to create an outflow pathway. The PBS was perfused to remove the erythrocytes and nonadherent leukocytes. Fluorescein‐conjugated concanavalin A (Con A, 50 μg/mL dissolved in PBS; Vector Laboratories, Burlingame, CA) was injected to label the adherent leukocytes for 5 min. The unbound lectin was then removed with PBS perfusion. The eyes were then enucleated and fixed in 4% paraformaldehyde before the retinas were dissociated and flat‐mounted. Leukostasis were observed using a Leica microscope (DM4000, Germany), and total adherent leukocytes were calculated (*n* = 6 retinas in each group).

### 2.7 Western blotting assay

The retina was harvested and homogenized in lysis buffer containing protease and phosphatase inhibitor mini tablets (Thermo Fisher Scientific, Waltham, MA). Nuclear expression of HMGB1 was detected in the nuclear fractions extracted by the NE‐PER Nuclear and Cytoplasmic Extraction Reagents (Thermo Fisher, No. 78833). The protein concentration was determined with bicinchoninic acid protein assay. Equal amounts of protein were loaded, and western blotting was performed as previously described.^29^ The band intensities were measured using the Image J software. Primary antibodies included ZO‐1 antibody (Invitrogen, 339 100), vascular endothelial‐cadherin antibody (Cell Signaling Technology, #2158), claudin‐5 antibody (Santa Cruz, sc‐374 221), (platelet derived growth factor receptor beta) PDGFR‐β antibody (Santa Cruz, sc‐374 573), HMGB1 antibody (Abcam), RAGE antibody (Abcam, ab30381), nuclear factor kappa B (NF‐κB) antibody (Abcam, ab32536), TLR4 antibody (Abcam, ab190377), tumor necrosis factor alpha (TNF‐α) antibody (Abcam, ab183218), interleukin (IL)‐1β antibody (Abcam, ab234437), and intercellular adhesion molecule 1 (ICAM‐1) antibody (Abcam, ab222736). β‐actin antibody (Abcam, ab6276) was used as the loading control. The assays were independently repeated three times for statistical analysis.

### 2.8 Electroretinogram

Electroretinogram was performed at the end of the sixth month after STZ induction. After overnight dark adaptation, the mice were anesthetized with zoletil and xylazine followed by pupil dilation using 1% tropicamide. Electroretinogram (ERG) was recorded by a Ganzfeld stimulator (Roland Consult, Germany). Scotopic ERG was recorded with a single flash of 1.3 ms duration with an intensity of 1.0 log cd s/m^2^. A total of 5 responses were documented and the average was calculated. The amplitudes of the ERG b‐wave were measured using the RETI system software and analyzed (*n* = 8 retinas from 8 mice in each group).

### 2.9 Terminal‐deoxynucleoitidyl transferase mediated nick end‐labeling (TUNEL) assay

The eyeballs were embedded in optimal cutting temperature compound overnight and sectioned. TUNEL staining (In Situ Cell Death Detection Kit, Fluorescein; Roche, IN, USA) was performed according to the manufacturer's instructions. The sections were imaged using a confocal microscope. For quantitative analysis, the TUNEL^+^ cell nuclei were counted in six retinas from six mice in each group.

### 2.10 Human retinal micrangium endothelial cell (HRMEC) culture and treatment

HRMECs were purchased from Procell Life Science&Technology (Wuhan, China). The HRMECs were grown on the poly‐L‐lysine‐coated plates in Dulbecco's Modified Eagle's Medium (DMEM) supplemented with 5% fetal bovine serum (FBS), 100 units/mL penicillin, 100 μg/mL streptomycin, endothelial cell growth factor, heparin, and hydrocortisone in a 5% CO_2_‐enriched atmosphere with constant humidity. To maintain uniform conditions, all experiments were performed using passage 5 HRMECs. HRMECs were grown in the 50 mM glucose medium (Sigma‐Aldrich) for 48 h. D‐Mannitol was used as an osmotic control. For the treatment, NK (1 μM and 2 μM) was pretreated for 2 h before HG stimulation. For HMGB1 silencing, siRNA constructs were diluted in buffer (Lipofectamine transfection reagent; Invitrogen Inc., Carlsbad, CA, USA) and transfected in culture medium of HRMECs at a working concentration of 100 nM for 24 h before NK treatment. Scrambled siRNA was used as control. The sequences for HMGB1‐siRNA were 5′‐GGC UUU CAC UUA AGA ACU UTT‐3′.

### 2.11 Transwell assay

The HRMECs were plated in a 24‐well plate. After trypsin digestion, DMEM containing 1% FBS was added to obtain a single‐cell suspension. The lower chamber was added with 800 μL of DMEM supplemented with 10% FBS. After 24‐h incubation, the HRMECs attached to the upper surface were scraped using a cotton swab, and the cells were fixed with 90% ethanol and stained with 0.1% crystal violet. The migrated cells were observed and counted under the microscope (Nikon, Tokyo, Japan). Six wells and three different fields per well were randomly chosen for further analysis.

### 2.12 Quantitative reverse transcription polymerase chain reaction and array analysis

The total RNA of HRMECs were extracted with TRIzol (Invitrogen) and converted into first‐strand cDNA using random hexamer primers and the Reverse Transcriptase Superscript II Kit (Invitrogen) according to the manufacturer's instructions. Polymerase chain reaction (PCR) was performed in a total volume of 20 mL containing 2 mL of cDNA, 10 mL of 2 × SYBR Premix Ex Taq, and 10 mmol/L of the primer pairs. The PCR amplification protocols consisted of 95°C for 30s and up to 40 cycles of 95°C for 5 s and 60°C for 34 s according to the manufacturer's instructions. The primers used for amplification were shown in Table S1. The assays were independently repeated three times for statistical analysis. For PCR arrays, the retinas were collected for the mouse quantitative PCR (qPCR) arrays (QIAGEN RT^2^ Profiler; SABioscience) analysis following the standard protocol, containing 48 genes related to angiogenesis and inflammatory cytokines. β‐actin and *Gapdh* were utilized as housekeeping genes for data normalization. Results were expressed as fold‐changes in gene expression.

### 2.13 Statistics

All experiments were repeated independently three times. The data were shown as mean ± SD. Two‐tailed Student's *t* test was used to compare the differences between two groups, and one‐way analysis of variance was carried out to compare multiple groups with post hoc analysis. *p* < .05 was considered as statistically significant.

## 3 RESULTS

### 3.1 NK ameliorated microvascular leakage in diabetic retinopathy

**FIGURE 1:**
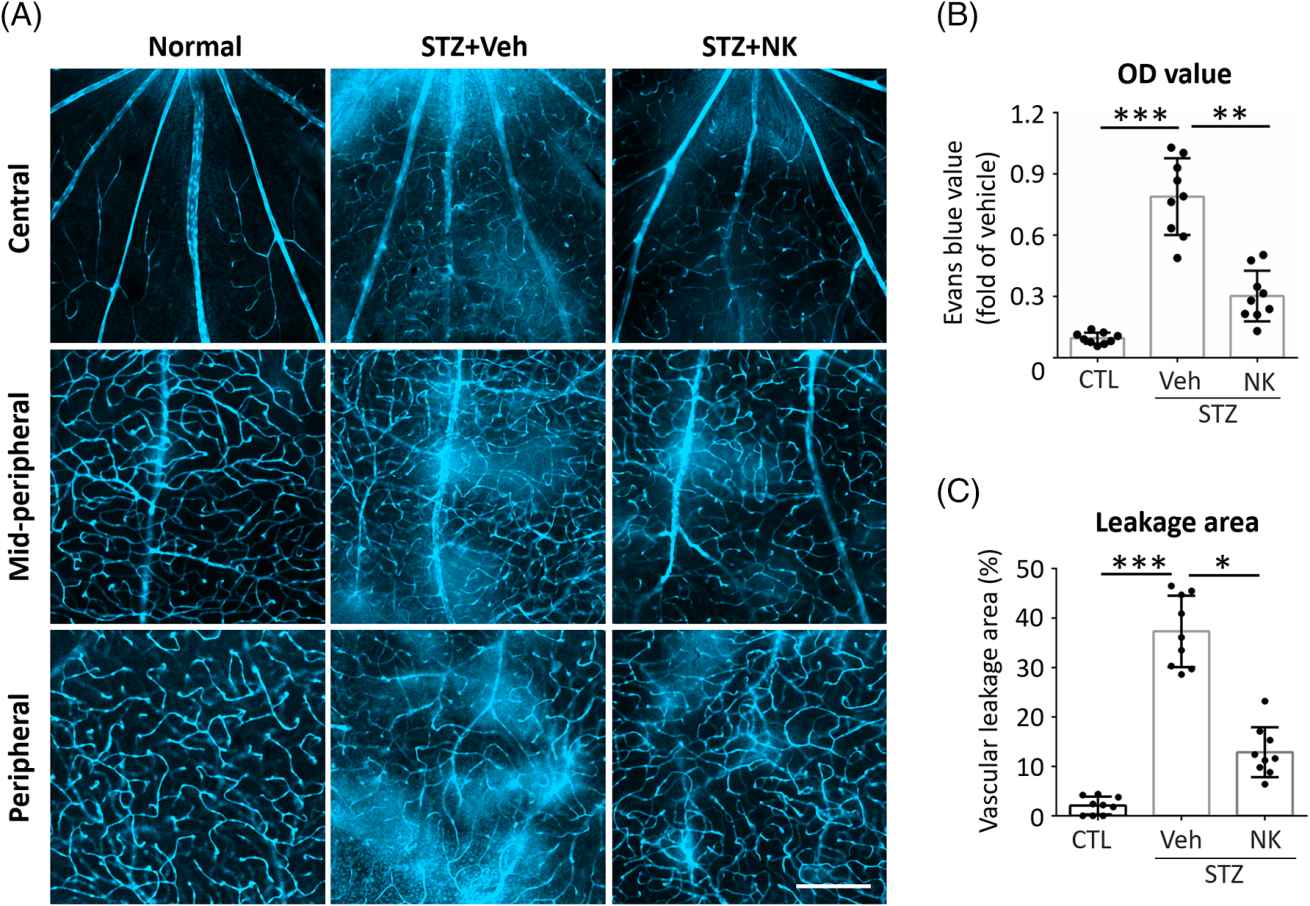
NK attenuated microvascular leakage in mouse models of diabetic retinopathy and ischemic retinopathy. (A–C) Representative images and statistical analysis of Evans blue assays of the STZ‐induced diabetic retinopathy model. In the diabetic retina, the breakdown of blood retinal barrier caused leakage of the dye into the retinal parenchyma, as compared to the presence of sharply outlined vessels in the normal retina. Treatment of NK (STZ + NK) markedly reduced vascular leakage as compared to the control treatment (STZ + Veh). *n* = 9 retinas in each group. Scale bar: 100 μm. Data are shown as mean ± SD. **p < .01. ***p < .001. CTL, control; NK, nattokinase; OD, optical density; STZ, streptozotocin; Veh, vehicle.

We first delineated the protective effect of NK against retinal microvascular leakage using the STZ‐induced diabetes model. NK was delivered by intravitreal injection bimonthly (Figure S1A). Successful modeling was confirmed by the elevated blood glucose levels (Figure S1B). On week 24, breakdown of the BRB presenting with Evans blue dye leakage was observed in the vehicle‐injected DR retinas, particularly in the midperipheral and peripheral areas. Of note, the retinas with intravitreal NK treatment exhibited distinct outline of the vessels with significantly less dye leakage (Figure 1A–C). These findings demonstrated an inhibitory effect of NK against retinal microvascular leakage.

### 3.2 NK improved high glucose‐induced retinal endothelial dysfunction

**FIGURE 2:**
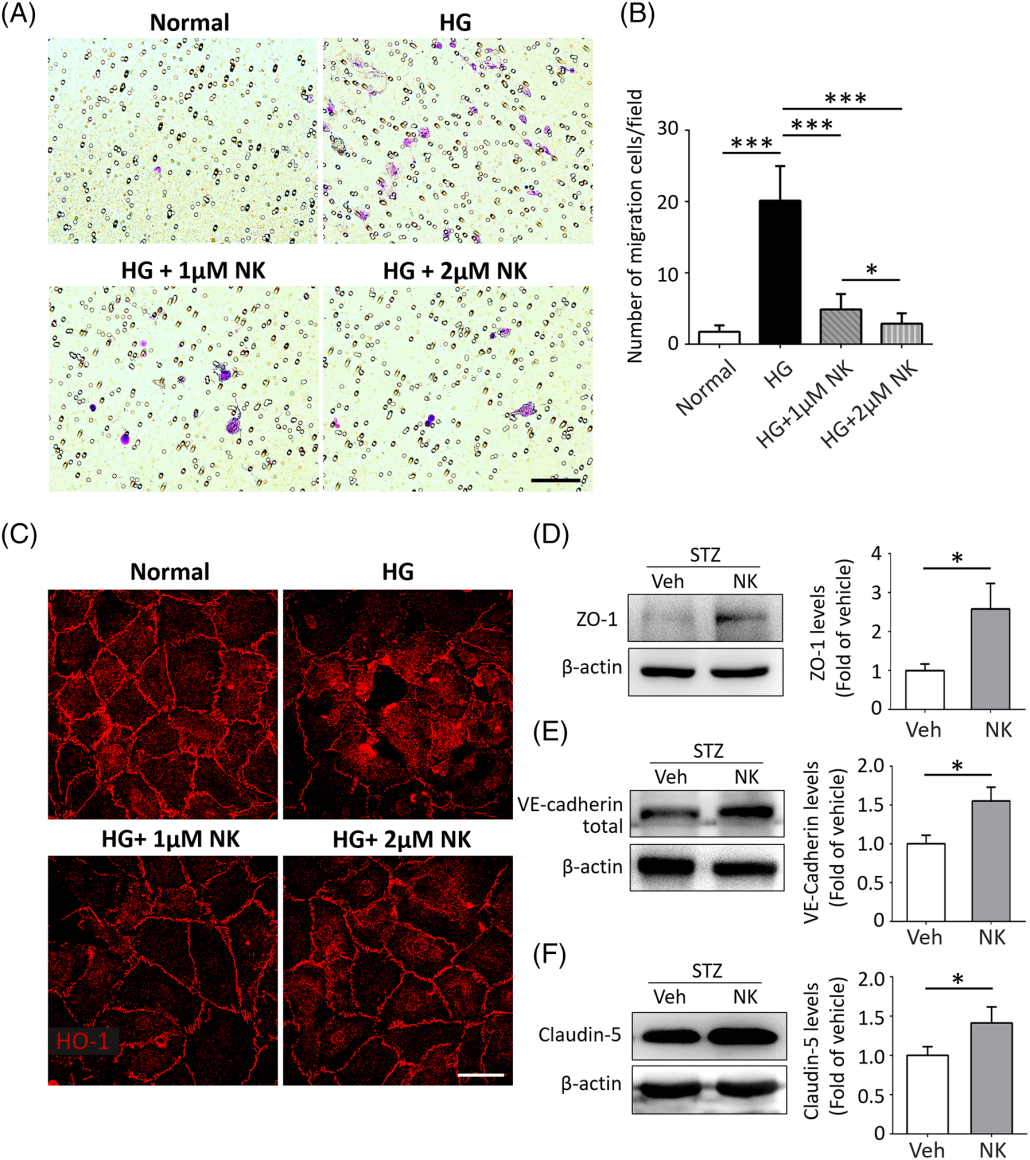
NK improved endothelial dysfunction via modulation of tight junctions. (A, B) Representative images and statistical analysis of transwell migration assays in cultured human retinal micrangium endothelial cells (HRMECs). High glucose (HG, 50 mM) increased HRMEC migration as compared to the normal glucose conditions, which was largely inhibited by pretreatment of NK (HG + NK) in a dose‐dependent manner. Purple, migrated cells; White, pores of the membrane. Scale bar: 100 μm. *N* = 6 wells and 3 fields per well were randomly chosen for analysis. (C) Representative images of the ZO‐1 immunostaining on HRMECs. NK improved the integrity of tight junction protein ZO‐1 under HG condition in a dose‐dependent manner as compared to the control treatment (Veh). Scale bar: 25 μm. (D–F) Western blotting results showed upregulated levels of endothelial junctional proteins, including ZO‐1, VE‐cadherin, and claudin‐5, in the STZ‐induced diabetic retina with NK treatment. β‐actin was used as the loading control. Data are shown as mean ± SD. *p < .05. ***p < .001. NK, nattokinase; STZ, streptozotocin; VE, vascular endothelial; Veh, vehicle.

HG has been demonstrated to cause dysfunction in vascular endothelial cells.^30^ We explored the in vitro effects of NK on HG‐induced HRMEC dysfunction using the transwell system. HG stimulation caused a significant increase in HRMEC migration, whereas pretreatment with NK effectively mitigated this effect in a dose‐dependent manner (Figure 2A, B). The vascular cell permeability was also evaluated by detecting the changes in tight junction structure and integrity illustrated by ZO‐1 staining. Discontinuous and disorganized structure of ZO‐1 were noted in HG‐treated HRMECs. Remarkably, pretreatment with NK strikingly improved ZO‐1 integrity in response to HG stimulation (Figure 2C). NK administration also induced upregulation of endothelial tight junction proteins, including ZO‐1 (Figure 2D), vascular endothelial‐cadherin (Figure 2E), and claudin‐5 (Figure 2F), in the STZ‐induced diabetic retinas as compared to the vehicle‐treated controls. Collectively, these data indicated a protective role of NK against HG‐induced endothelial dysfunction.

### 3.3 NK prevented pericyte loss in the diabetic retina

**FIGURE 3:**
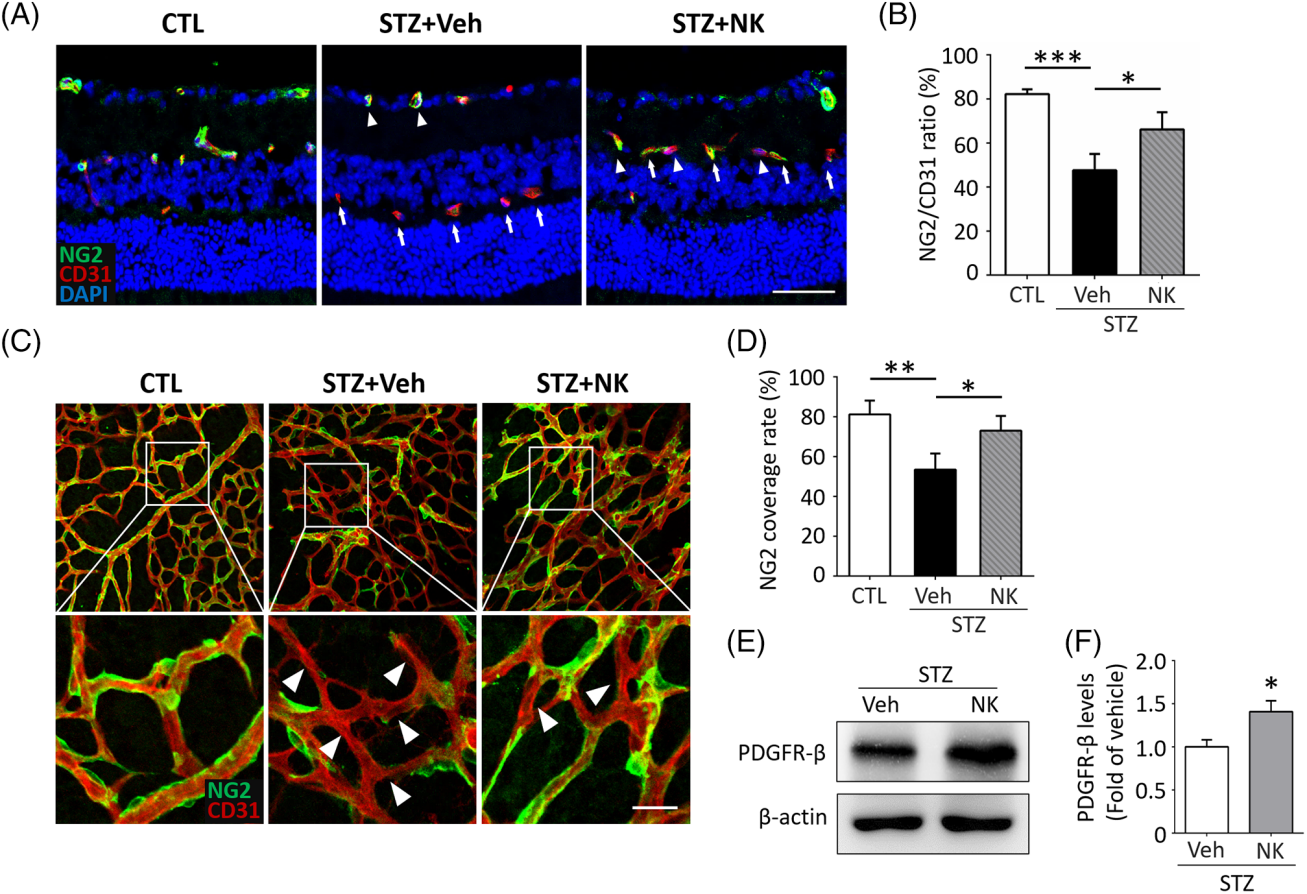
NK prevented pericyte loss in the STZ‐induced diabetic retina. (A, B) Representative images and statistical analysis of coimmunostaining of CD31 and NG2 on retinal cryosections. In normal retina, the vessel integrity was maintained as most of CD31^+^ endothelial cells were covered with NG2^+^ pericytes, whereas STZ‐induced diabetes resulted in an increase in the number and proportion of CD31^+^NG2^−^ cells (arrows), indicating the loss of pericyte coverage of vessels. NK treatment significantly increased the number and percentage of CD31^+^NG2^+^ cells (arrowheads) as compared to the control treatment (Veh). *n* = 6 retinas from 6 mice for analysis. Scale bar: 100 μm. (C, D) Representative images and statistical analysis of co‐immunostaining of CD31 and NG2 on retinal flat‐mounts. Arrowheads indicated the microvessels with pericyte loss, which was partly rescued by treatment of NK as compared to the control treatment (Veh). Scale bar: 50 μm. (E, F) Western blotting analysis showed upregulation of PDGFR‐β, a pericyte marker, in the NK‐treated diabetic retina. β‐Actin was used as the loading control. Data are shown as mean ± SD. *p < .05. **p < .01. ***p < .001. CTL, control; NG2, nerve/glial antigen 2; NK, nattokinase; PDGFR‐β, platelet derived growth factor receptor beta; STZ, streptozotocin; Veh, vehicle.

Loss of pericytes plays an essential role in promoting endothelial dysfunction and BRB breakdown.^7^ We next investigated the protective potential of NK on pericyte loss. The diabetic retinas showed reduced number of pericytes labeled with NG2 accompanied by an increased proportion dysfunctional capillaries that lacked NG2 expression but displayed CD31 positivity, whereas NK treatment significantly reversed this process (Figure 3A, B). Consistently, dropouts of pericytes under the diabetic condition were also observed using the flat‐mounted retinas, presenting with increased number of endothelial cells with incomplete coverage of pericytes, which was largely abolished by NK administration (Figure 3C, D). In addition, PDGFR‐β, a pericyte marker, was also upregulated in the retina by NK treatment (Figure 3E, F), further indicating the potential role of NK in rescuing pericytes loss in the diabetic retinas.

### 3.4 NK attenuated diabetes‐stimulated gliosis and inflammatory responses

**FIGURE 4:**
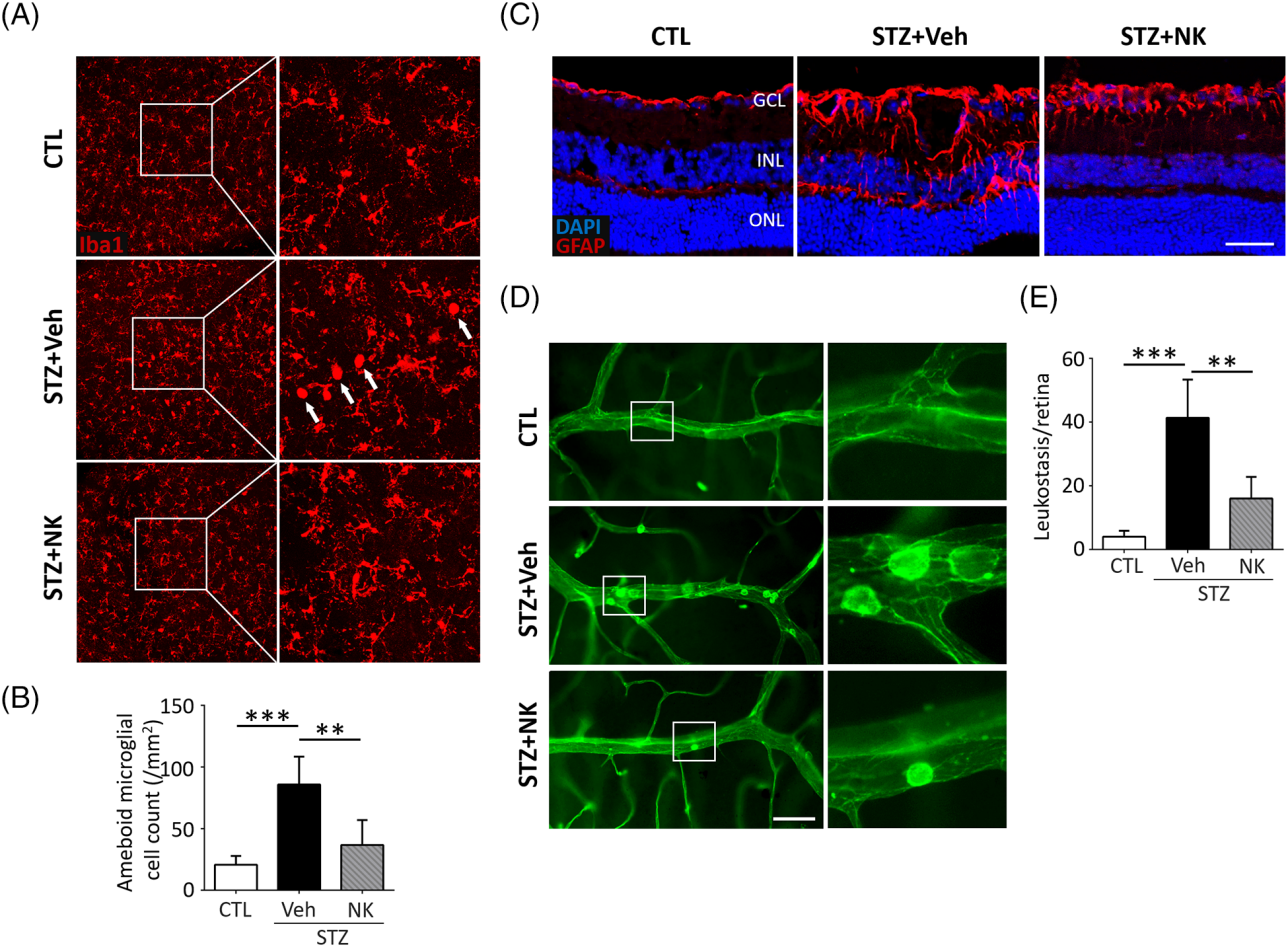
NK orchestrated glial activation and alleviated leukostasis in the STZ‐induced diabetic retina. (A, B) Representative images and statistical analysis of immunostaining of the Iba1^+^ microglia on retinal whole mounts. Microglia presented an activated “ameboid” profile, featured with enlarged somas and short lamellipodia (arrows) in the diabetic retinas as compared to a ramified microglia phenotype that presented small cell bodies and long processes in the normal retinas (CTL). NK treatment reduced the number of ameboid microglia in the diabetic retinas as compared to the control treatment (Veh). *n* = 6 retinas from 6 mice for analysis. Scale bar: 50 μm. (C) Immunostaining of GFAP on retinal astrocytes and Müller cells. In the normal retina, GFAP was mainly localized to astrocytes in the superficial layer. In the diabetic retina, intensive GFAP labeling in astrocytes and Müller cells in the inner layer was detected, whereas NK significantly reduced GFAP immunoreactivity as compared to the control treatment (Veh). Scale bar: 100 μm. (D, E) Representative images and statistical analysis of leukostasis assay showing increased number of adhered leukocytes in the STZ‐induced diabetic retina, which was abolished by NK treatment, as compared to the control treatment (Veh). *n* = 6 retinas from 6 mice for analysis. Scale bar: 50 μm. Data are shown as mean ± SD. *p < .05, **p < .01. ***p < .001. CTL, control; GFAP, glial fibrillary acidic protein; Iba1, ionized calcium‐binding adapter molecule 1; NK, nattokinase; STZ, streptozotocin; Veh, vehicle.

NK has been demonstrated to possess potent anti‐inflammatory and immunoregulatory effects in experimental models of Alzheimer's disease.^23^ To investigate the potential alleviation of glial activation in the diabetic retina by NK, we performed immunostaining of Iba1 and GFAP, which are markers for microglia and astrocytes/Müller cells, respectively. Increased number of activated microglia with an “ameboid‐shape” phenotype, characterized by enlarged somas and short lamellipodia, was observed in the vehicle‐treated diabetic retina, as compared to the normal retina, where microglia displayed small cell bodies and long processes NK administration resulted in decreased proportion of activated microglia (Figure 4A, B). In addition, the enhancement in the GFAP immunoreactivity, indicating reactive gliosis in astrocytes and Müller cells within the diabetic retina, was reversed by NK treatment (Figure 4C). To evaluate inflammatory responses, we performed a leukostasis assay, which revealed that NK significantly ablated the leukocyte adhesion in diabetic microvessels (Figure 4D, E). These data provide evidence for the protective effects of NK against reactive gliosis and neuroinflammation in DR.

### 3.5 HMGB1 signaling was responsible for NK‐mediated vasoprotection and anti‐inflammation

**FIGURE 5:**
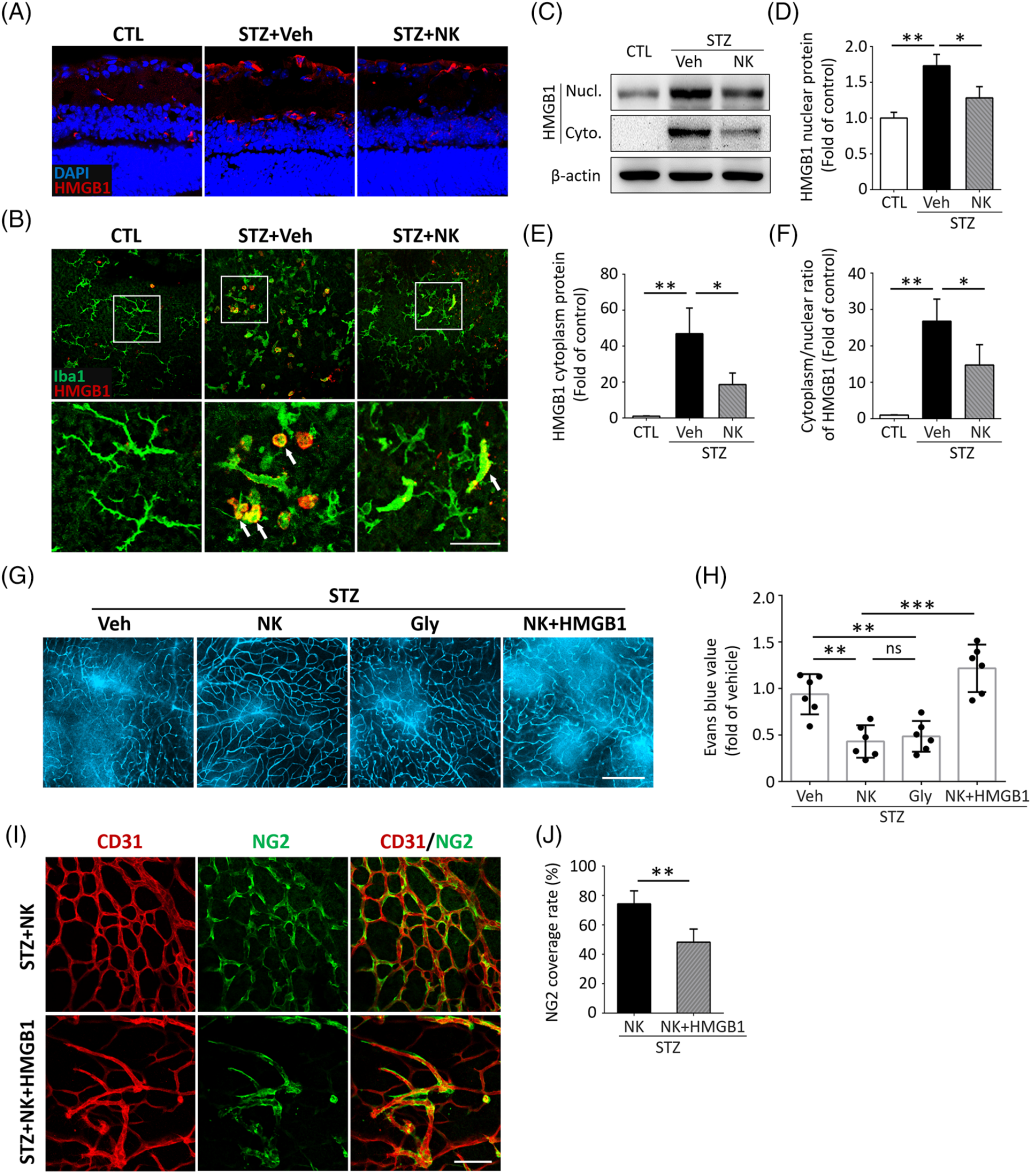
HMGB1 was involved in the NK‐mediated protective effect against experimental diabetic retinopathy. (A) Representative images and statistical analysis of immunostaining assay showed an increased fluorescence intensity of HMGB1 in the diabetic retina, which was markedly reversed by NK administration, as compared to the control treatment (Veh). *n* = 6 retinas from 6 mice for analysis. Scale bar: 50 μm. (B) Double immunostaining assay showed that HMGB1 was mainly colocalized with the ameboid microglia. (C–F) Western blotting analysis using the NE‐PERTM Nuclear and Cytoplasmic Extraction Reagents showed that NK inhibited the cytoplasmic protein levels and nuclear‐cytoplasmic translocation of HMGB1. (G, H) Evans blue assay in retinal whole mounts showed comparable vasoprotective effect of NK and glycyrrhizin (Gly), a specific HMGB1 inhibitor, in the diabetic retina. Coadministration of recombinant HMGB1 abolished the effect of NK against vascular leakage. *n* = 6 retinas in each group. Scale bar: 100 μm. (I‐J) The prosurvival effect of NK on NG2^+^ pericytes was reduced by cotreatment of recombinant HMGB1. Scale bar: 100 μm. Data are presented as mean ± SD. *p < .05, **p < .01, ***p < .001. CTL, control; Cyto, cytoplasmic; HMGB1, high mobility group box 1; NG2, nerve/glial antigen 2; NK, nattokinase; Ns, no significance; Nucl, nuclear; STZ, streptozotocin; Veh, vehicle.

To investigate the molecular signaling involved in NK‐mediated microvascular protection, we conducted a qPCR‐based microarray assay and found that HMGB1, a key inflammatory mediator, exhibited the highest upregulation in the diabetic retina compared to the nondiabetic controls (Figure S2A). NK treatment impaired the production of HMGB1, as well as other angiogenic and inflammatory factors (Figure S2B). Consistent with the qPCR results, we observed an increase in HMGB1 expression in the diabetic retina, which was reduced by NK treatment (Figure 5A). To define the cellular source of HMGB1, we performed double immunostaining of HMGB1 with different retinal cell types, which showed that the HMGB1 expression colocalized with microglia, retinal ganglion cells, vascular endothelial cells, pericytes, and astrocytes, with microglia being the primary source (Figure 5B, Figure S3). Interestingly, HMGB1 was abundant in activated ameboid microglia in the diabetic retina, and NK significantly reduced its expression (Figure 5B). Because HMGB1 translocates from the nucleus to the cytoplasm during cell activation, we detected the changes in HMGB1 protein levels in different cellular locations and found that NK treatment significantly inhibited cytoplasmic protein levels and the nucleo‐cytoplasmic translocation of HMGB1 (Figure 5C–F). It was also noted that the inhibitory effect of NK on vascular leakage in the diabetic retina was comparable with glycyrrhizin, a known HMGB1 inhibitor, and that recombinant coadministration of HMGB1 abolished the protective effect of NK against vascular leakage (Figure 5G, H), further highlighting the involvement of HMGB1 reduction in NK treatment. Finally, the rescuing effect of NK in pericyte loss was also reversed by cotreatment with HMGB1, indicating the role of HMGB1 reduction in NK treatment (Figure 5I, J).

**FIGURE 6:**
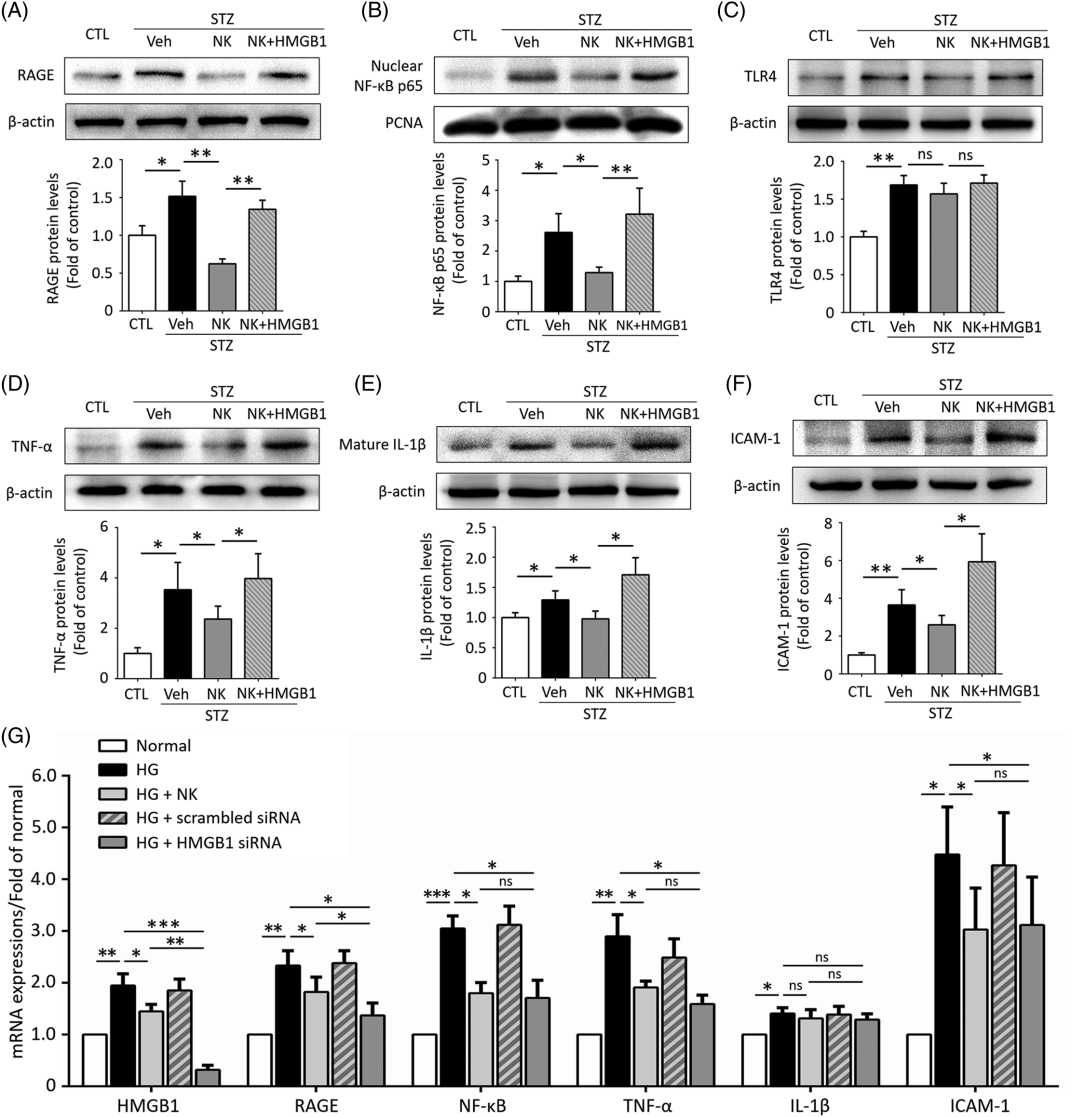
NK modulated HMGB1/RAGE/NF‐κB signaling and its downstream inflammatory cytokines in the diabetic retina and cultured HRMECs. (A–F) Representative images of western blotting assays and statistical analysis. NK treatment down‐regulated the levels of RAGE (A) and NF‐κB (B) downstream of HMGB1, but not toll‐like receptor 4 (TLR4) (C), in the diabetic retina, as compared to the control treatment (Veh). NK treatment significantly reduced the expression of several pro‐inflammatory cytokines, including TNF‐α (D), IL‐1β (E), and ICAM‐1 (F), as compared to the control treatment (Veh). Proliferating cell nuclear antigen (PCNA) was used as the loading control for nuclear NF‐κB p65 expression. (G) qPCR analysis for the HMGB1 signaling and its downstream molecules in cultured HRMECs. Both NK and HMGB1 siRNA treatment could downregulated the levels RAGE/NF‐κB and pro‐inflammatory cytokines, except for IL‐1β. The western blotting and qPCR assays were independently repeated for three times in the analysis. Data are shown as mean ± SD. *p < .05, **p < .01, ***p < .001. CTL, control; HG, high glucose; HMGB1, high mobility group box 1; HRMEC, human retinal micrangium endothelial cell; ICAM‐1, intercellular adhesion molecule 1; IL‐1β, interleukin‐1β; NF‐κB, nuclear factor kappa B; NK, nattokinase; Ns, no significance; qPCR, quantitative polymerase chain reaction; RAGE, receptor for advanced glycation end‐products; STZ, streptozotocin; TNF‐α, tumor necrosis factor‐alpha; Veh, vehicle.

We next explored the downstream signaling molecules of HMGB1 in the NK‐treated diabetic retina. Western blot analysis showed that NK administration significantly downregulated the RAGE and its downstream NF‐κB but not TLR4. Besides, the protective effects of NK were largely reversed when cotreated with HMGB1 (Figure 6A–C). NK also dampened the expression of several proinflammatory cytokines downstream of RAGE/NF‐κB, including TNF‐α, mature IL‐1β, and ICAM‐1, which could also be reversed by HMGB1 cotreatment (Figure 6D–F). Similar findings were observed at the mRNA levels in HG‐stimulated HRMECs. NK and HMGB1 siRNA exhibited comparable effects in inhibiting the RAGE/NF‐κB axis and downstream cytokines, with the exception of IL‐1β (Figure 6G). Collectively, these data indicated that NK exerts vasoprotective and anti‐inflammatory functions, at least in part, by modulating the HMGB1 signaling.

### 3.6 Neuroprotective effects of NK against diabetic retinal injury

**FIGURE 7:**
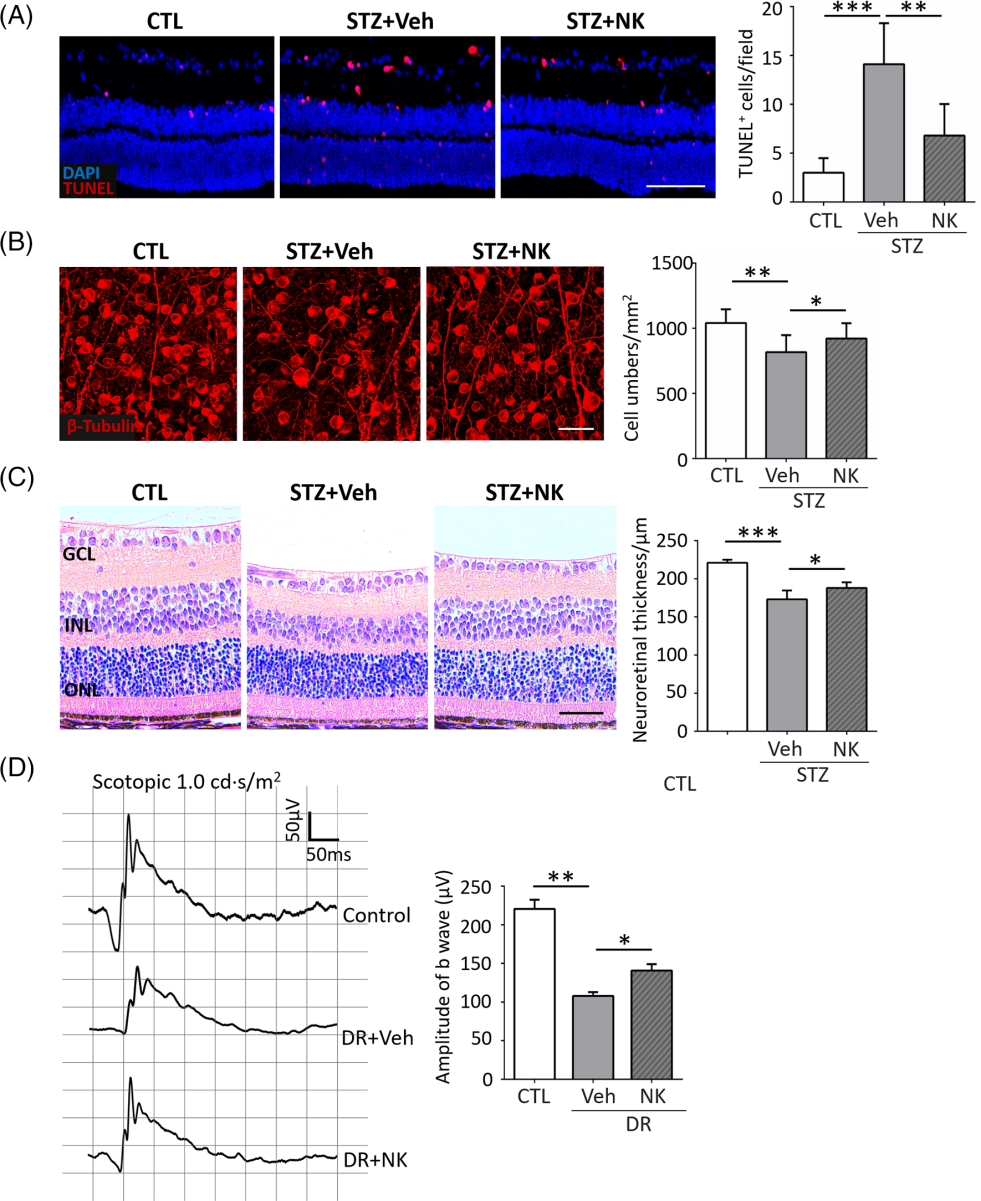
NK rescued retinal cells from apoptosis and improved neural functions in the diabetic retina. (A) TUNEL assay on retinal cryosections showed that NK significantly prevented retinal neurons from apoptosis as compared to the control treatment (Veh). Scale bar: 100 μm. *n* = 6 retinas from 6 mice for analysis. (B) Immunostaining of β‐tubulin on retinal whole mounts showed that NK increased the number of retinal ganglion cells under diabetic conditions. Scale bar: 50 μm. (C) Representative and statistical analysis of H&E staining. The decrease in neuroretinal thickness in DR mice was partially rescued by NK treatment. Scale bar: 100 μm. (D) Representative and statistical analysis of ERG in normal and DR mice. The amplitudes of b wave in the NK‐treated mice significantly increased as compared to that of the vehicle‐treated mice (Veh). *n* = 9 retinas from 9 mice in each group. Data are shown as mean ± SD. *p < .05, **p < .01, ***p < .001. CTL, control; DR, diabetic retinopathy; ERG, electroretinogram; H&E, hematoxylin and eosin; NK, nattokinase; STZ, streptozotocin; TUNEL, terminal‐deoxynucleoitidyl transferase mediated nick end‐labeling; Veh, vehicle.

While exerting inhibitory effects on diabetic microvasculopathy and neuroinflammation, NK was found to exhibit neuroprotective properties. As shown in Figure 7A, NK treatment reduced the number of apoptotic cells within the inner retina. NK also rescued retinal ganglion loss (Figure 7B) and increased the neuroretinal thickness (Figure 7C) in the diabetic retinas. In addition, mice treated with NK presented enhanced amplitudes of b waves in ERG recording compared to the vehicle‐treated controls, indicating an improvement in visual function (Figure 7D).

## 4 DISCUSSION

In recent decades, there has been a predominant focus on VEGF‐based therapies and the management of neovascular complications in advanced DR, whereas strategies targeting microvascular impairment in early‐stage disease have yet to be explored. There is an ongoing need for effective, safe, and cost‐efficient pharmacologic approaches targeting the pathological mechanisms of early DR events. Discovered in 1987, NK, the most active ingredient found in natto,^31^ demonstrates multiple beneficial effects on cardiovascular health and has been pursued as a promising alternative agent.^20^ In this study, we revealed novel protective effects of NK against microvascular leakage and neuroinflammation in an experimental DR model, suggesting a promising potential of this natural product in the treatment of DR.

Pericyte loss plays a key role in the progression of early‐stage DR, which influences the capillary remodeling, endothelial dysfunction, and disruption of interendothelial tight junctions, ultimately resulting in microvascular leakage and pathological angiogenesis.^32^, ^33^ Previous studies have focused on the biochemical pathways involved in retinal pericyte dropouts, including oxidative protein Low‐density lipoprotein‐induced injury,^34^ immune attack mediated by autoantibodies,^35^ and the toxicity of advanced glycation end products.^36^ However, the exact mechanisms underlying these processes are not yet fully understood. In this study, our data confirm that NK treatment inhibits HMGB1‐mediated pericyte loss in the diabetic retina. Indeed, overexpression and cytoplasmic translocation of HMGB1 have been strongly associated with the inflammation cascade and neural cell death in DR pathology,^37^ and intravitreal injection of HMGB1 could replicate early DR events, such as increased vascular permeability and disruption of tight junctions.^38^ HMGB1 has also been found to disrupt the migration of cultured vascular endothelial cells through activation of signal transducer and activator of transcription 3.^39^ Therefore, the downregulation of HMGB1 and its inflammatory signaling pathways may in part explain the protective effect of NK against diabetic microvasculopathy and neuroinflammation.

Although a link between HMGB1 and pericyte dropouts has been proposed, HMGB1 stimulation alone may not directly induce significant pericyte loss.^40^ In line with this, we did not observe overexpression of HMGB1 in pericytes but rather in ameboid microglia, which are resident immune cells in the retina. This suggests the involvement of microglia in HMGB1‐mediated pericyte death. Importantly, NK treatment not only reduced the levels of HMGB1 but also decreased the number of ameboid microglia, indicating its potential for immunoregulation in the diabetic retina.

NK has recently demonstrated to exert anti‐inflammatory and antiapoptotic activities.^20^ NK supplementation significantly delays the neurodegenerative process in animal models of Alzheimer's disease by decreasing the levels of IL‐6 and p53 while increasing B‐cell lymphoma 2 expression in brain neurons.^23^ Moreover, NK exhibited robust protective effects against lipopolysaccharide‐induced acute kidney injury through modulating the inflammation cascade and oxidative stress.^24^, ^41^ In this study, the administration of NK caused a remarkable reduction in proinflammatory cytokines, such as TNF‐α, IL‐1β, and ICAM‐1, in the diabetic retina. In addition, the anti‐inflammatory effect was largely abolished when recombinant HMGB1 was costimulated, indicating that NK treatment suppressed HMGB1 in the diabetic retina.

In this study, NK was delivered via bimonthly intravitreal injection. Intravitreal administration of NK did not induce significant retinal toxicity in retinal cells and vessels.^42^ Although intravitreal administration allows for a higher local concentration and minimizes systemic side effects, it remains an invasive treatment approach with the risk of infection. Exploring the feasibility of NK treatment via oral administration is important. Unfortunately, our preliminary data using the STZ‐induced diabetes model did not provide substantial evidence for the beneficial effect of oral intake of NK (data not shown). Possible explanations include a relatively low local concentration and short‐term delivery of the product. Further investigations are needed to explore the protective effect of long‐term oral NK supplementation in animal models and even in patients with DR.

One limitation of this study is the lack of direct evidence for the critical role of HMGB1‐induced microglia toxicity in DR with NK treatment, as NK may also regulate HMGB1 expression in other cell types. The development of conditional knockout of HMGB1 in microglia would be ideal to address this issue. In addition, there might be other signaling molecules involved, as suggested by the qPCR array, in the process of NK treatment, which are yet to be explored.

The urgent need for effective, safe, and cost‐efficient pharmacologic approaches to treat early DR is evident. The current study demonstrated the protective roles of NK against microvascular damage and neuroinflammation in a STZ‐induced DR model, in part through modulation of the HMGB1 signaling pathway. Although NK still presents challenges in terms of being suitable for human DR treatment, such as maintaining protease activity, determining optimal dosage, and selecting the appropriate delivery route, our research provides preliminary evidence for the therapeutic potential of NK in diabetic retinal vascular diseases. Given that NK is considered a valuable dietary supplement or nutraceutical, it is of interest and importance that future clinical studies investigate the treatment effects of oral NK supplementation on DR, as well as other retinal vascular diseases.

## AUTHOR CONTRIBUTIONS

Zijing Huang contributed to the conception and design of the experiments. Zijing Huang, Shaolang Chen, Jiajian Lang, Chong‐Bo Chen, and Yanxuan Xu were responsible for data collection, analysis, and interpretation. Zijing Huang drafted the article. Tsz Kin Ng, Qingping Liu, Weiqi Chen, and Dingguo Huang contributed to the data analysis. Wai Kit Chu revised the article and gave supervision. All authors approved the final version of the manuscript for submission.

## FUNDING INFORMATION

This work was supported by the National Natural Science Foundation of China (No. 82101112) and the Special Support Plan for High‐Level Talents in Guangdong Province for Young Top Talents in Science and Technology Innovation.

## CONFLICT OF INTEREST STATEMENT

The authors declare that they have no competing interests.

## Supporting information


**Figure S1:** The establishment and treatment of STZ‐induced diabetic retinopathy mice.

**Figure S2:** qPCR array showing RNA levels of inflammatory and angiogenic molecules.

**Figure S3:** Expression of HMGB1 in various cell types of the diabetic retina.

**Table S1.** PCR primers in this study.

**Figures S1‐S3.** Descriptions.

## Data Availability

The data sets generated during and/or analyzed during the current study are available from the corresponding author on reasonable request.
