# Supplementary figures and images for: Protective effects of nattokinase against microvasculopathy and neuroinflammation in diabetic retinopathy
Source: J Diabetes. 2023 Jul 4;15(10):866–80. doi: 10.1111/1753-0407.13439 (PMC10590680; doi:10.1111/1753-0407.13439)

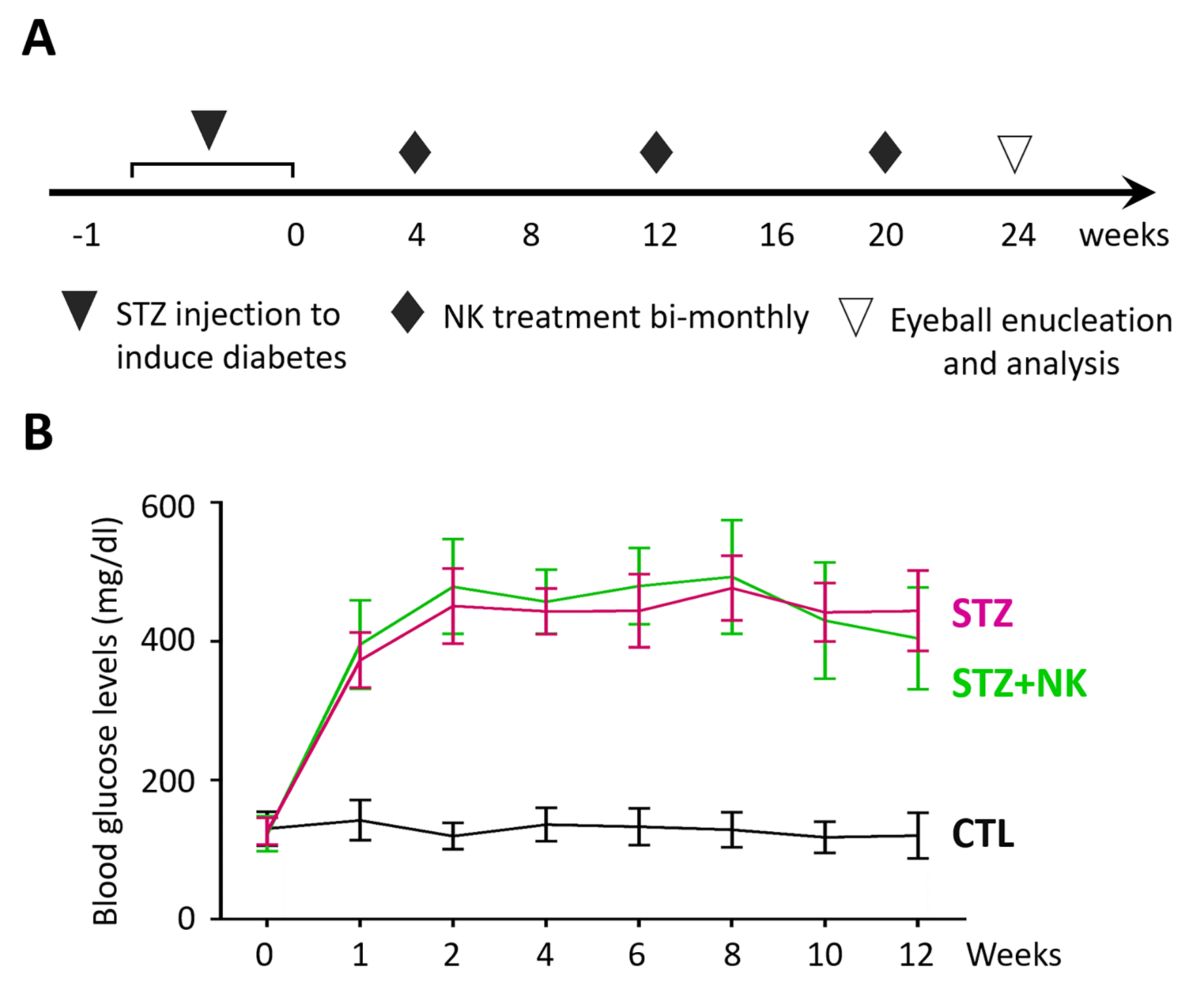

Supplement: Supplementary file 1 — Figure S1: The establishment and treatment of STZ‐induced diabetic retinopathy mice. [file JDB-15-866-s004.tif]

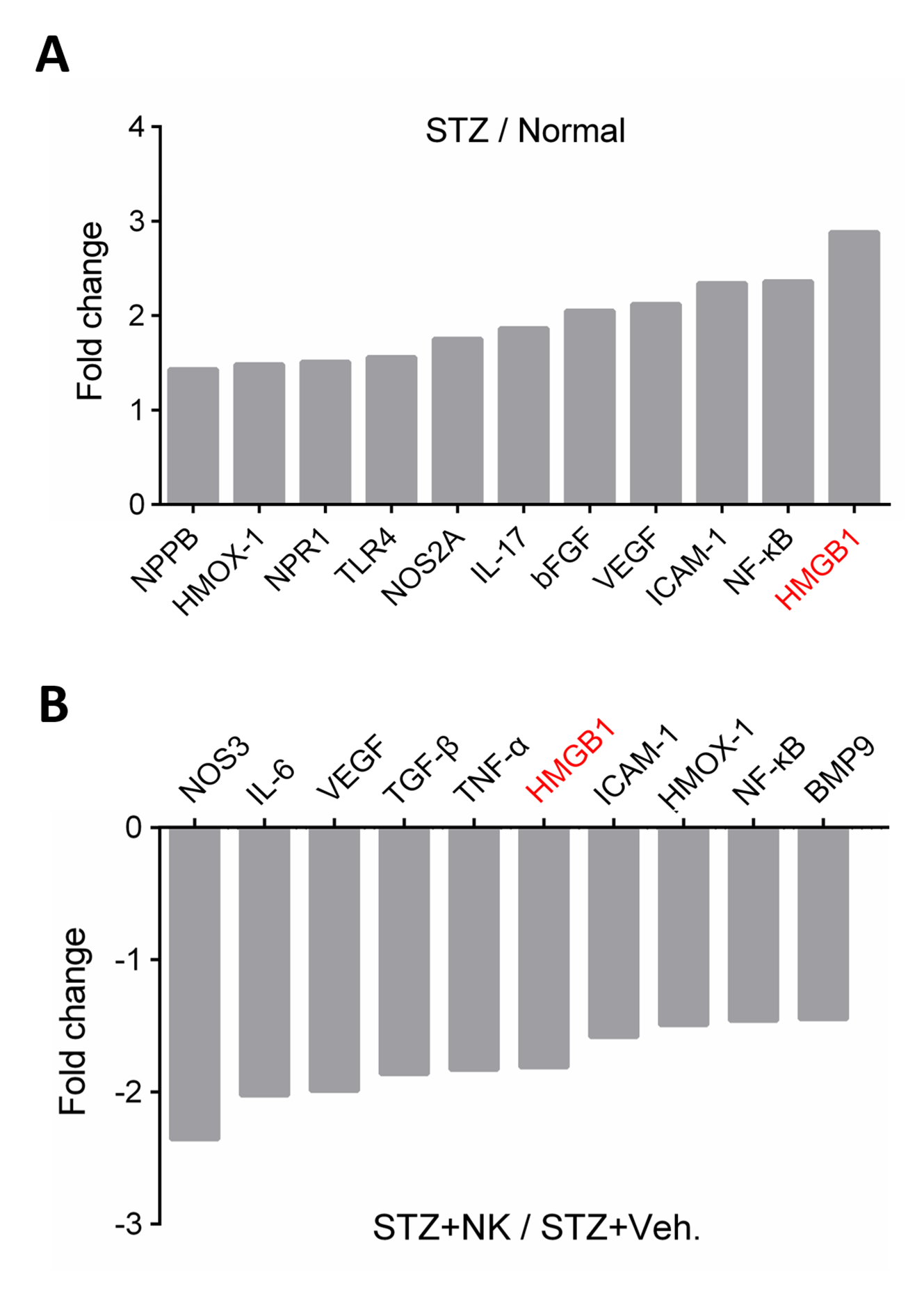

Supplement: Supplementary file 2 — Figure S2: qPCR array showing RNA levels of inflammatory and angiogenic molecules. [file JDB-15-866-s005.tif]

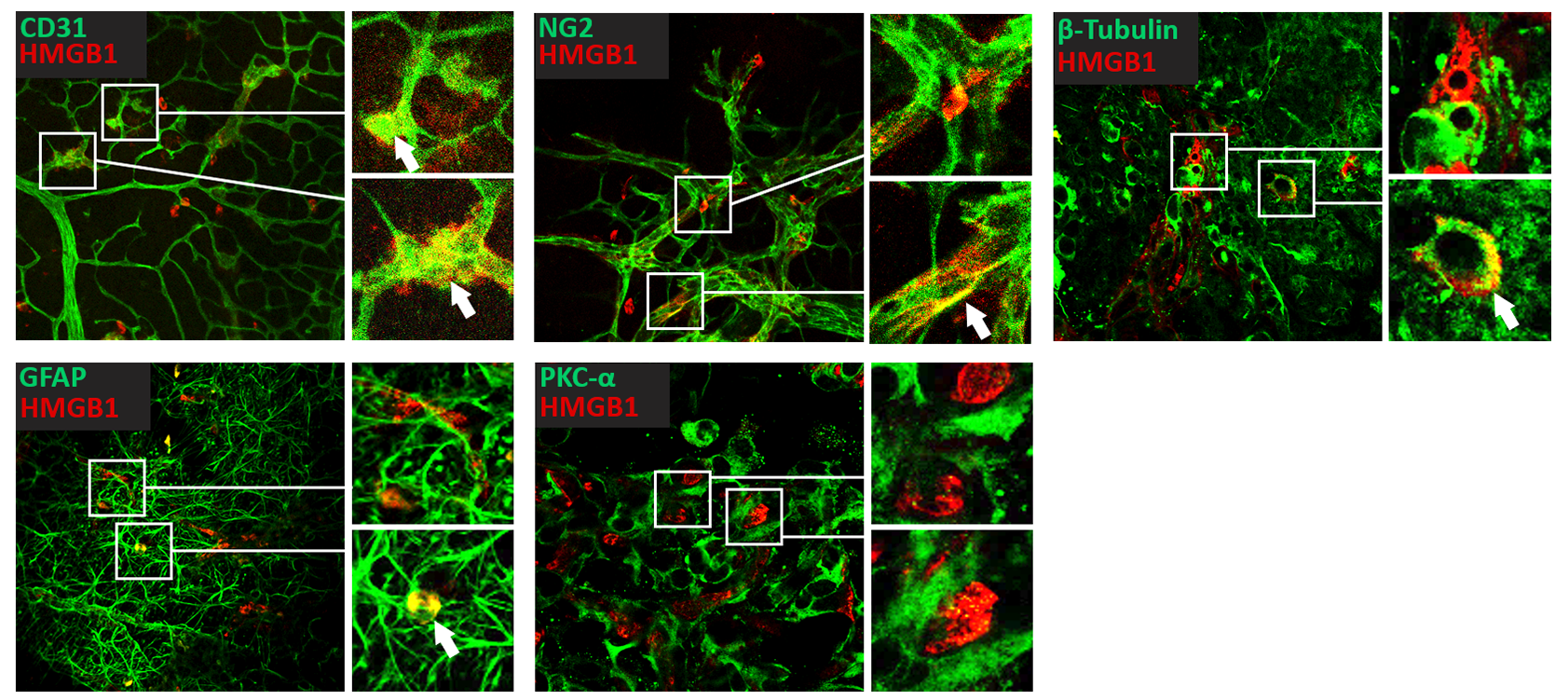

Supplement: Supplementary file 3 — Figure S3: Expression of HMGB1 in various cell types of the diabetic retina. [file JDB-15-866-s001.tif]
