# Supplementary material for: Protective effects of nattokinase against microvasculopathy and neuroinflammation in diabetic retinopathy
Source: J Diabetes. 2023 Jul 4;15(10):866–80. doi: 10.1111/1753-0407.13439 (PMC10590680; doi:10.1111/1753-0407.13439)
Supplement: Supplementary file 4 — Table S1. PCR primers in this study. [file JDB-15-866-s003.docx]

Supplementary Table 1. PCR primers in this study.

| Gene name | Orientation | Primer sequence (5’ to 3’) |
| --- | --- | --- |
| *hmgb1* | Forward | TAT GGC AAA AGC GGA CAA GG |
|  | Reverse | CTT CGC AAC ATC ACC AAT GGA |
| *rage* | Forward | ATT GGT GGT GGA GCC AGA AG |
|  | Reverse | GGC ACA CCA TCC TTC ATC CA |
| *nf-κb* | Forward | ATG TGG AGA TCA TTG AGC AGC |
|  | Reverse | CCT GGT CCT GTG TAG CCA TT |
| *tnf-α* | Forward | GAG GCC AAG CCC TGG TAT G |
|  | Reverse | CGG GCC GAT TGA TCT CAG C |
| *il-1β* | Forward | TCA GGC AGA TGG TGT CTG TC |
|  | Reverse | GGT CTA TAT CCT CCA GCT GC |
| *icam-1* | Forward | CCG GAA GGT GTA TGA ACT G |
|  | Reverse | CAG TTC ATA CAC CTT CCG G |
