# Supplementary material for: Protective effects of nattokinase against microvasculopathy and neuroinflammation in diabetic retinopathy
Source: J Diabetes. 2023 Jul 4;15(10):866–80. doi: 10.1111/1753-0407.13439 (PMC10590680; doi:10.1111/1753-0407.13439)
Supplement: Supplementary file 5 — Figures S1‐S3. Descriptions. [file JDB-15-866-s002.docx]

**Supplementary Figure 1. The establishment and treatment of STZ-induced diabetic retinopathy mice.** (A) The protocol showing the establishment of STZ-induced diabetic retinopathy murine model and treatment of NK. (B) Blood glucose levels of STZ-induced diabetic mice treated with (STZ+NK) or without (STZ) NK and controls (CTL).

**Supplementary Figure 2. qPCR array showing RNA levels of inflammatory and angiogenic molecules.** (A) Enrichment of inflammatory and angiogenic genes expression in the STZ-induced diabetic retinas over non-diabetic retinas. n = 6 retinas in each group. (B) NK treatment down-regulated the mRNA expression of inflammatory and angiogenic genes in the diabetic retinas. n = 6 retinas in each group.

**Supplementary Figure 3. Expression of HMGB1 in various cell types of the diabetic retina.** Double immunostaining of HMGB1 together with the followings was performed: CD31, a marker for vascular endothelial cells; NG2, a marker for pericytes; β-Tubulin, a marker for retinal ganglion cells; GFAP, a marker for astrocytes and Müller cells; PKC-α, a marker for bipolar cells. HMGB1 was co-localized with part of retinal ganglion cells, vascular endothelial cells, pericytes, and astrocytes and Müller cells (arrows).
